# Pediatric ACEs and Related Life Events Screener (PEARLS v2): Adaptations and Acceptability

**DOI:** 10.1007/s40653-025-00774-2

**Published:** 2025-11-05

**Authors:** Meghan C. Evans, Maryam Kia-Keating, Danielle Hessler, Daniela Sarmiento Hernández, Marina Schechter, Daniel Correa-Bucio, Sarah Ismail, Lisa James, Dayna Long, Neeta Thakur

**Affiliations:** 1 Department of Counseling, Clinical, and School Psychology, University of California, Santa Barbara, CA USA; 2 Departments of Family and Community Medicine, University of California, San Francisco, CA USA; 3 Department of Medicine, University of California, San Francisco, CA USA; 4 Department of Pediatrics, University of California, San Francisco, CA USA; 5 UCSF Benioff Children’s Hospital Oakland, Oakland, CA USA; 6 California ACEs Learning and Quality Improvement Collaborative (CalQIC), San Francisco, CA USA; 7 Futures without Violence, San Francisco, CA USA

**Keywords:** Adverse childhood experiences, PEARLS, Screening, Pediatrics

## Abstract

Due to a demonstrated relationship between Adverse Childhood Experiences (ACEs) and a range of psychological, physical, and social risks, there has been a growing movement to integrate routine ACEs screening in pediatric settings. As screenings have increased, there has also been a call for research to uncover areas for improvement in the implementation of these screenings. The Pediatric ACEs and Related Life Events Screener (PEARLS) is one common tool used for screening that has demonstrated construct validity in identifying three domains of adversity: maltreatment, household challenges, and social context. A second version of PEARLS (PEARLS v2) was adapted and refined in response to feedback collected from public health professionals, physicians, clinical psychologists, mental health specialists, and patients in a variety of settings, including clinics, state advisory boards, and health equity counsels. To ensure acceptability, this study examines caregiver feedback on PEARLS v2 through semi-structured interviews with 13 caregivers of child patients in Federally Qualified Health Centers. Using Rapid Qualitative Analysis, results highlight caregiver feedback on the addition of a written introduction to the screener and overall implementation preferences (i.e., location, length of time). Results also underscore caregiver feedback and suggestions on item clarity and acceptability, the addition of strength-based questions, perspectives on caregiver honesty in completing the screener, and preferences regarding provider response and resources. Findings support PEARLS v2 adaptations and provide caregiver-driven feedback to address acceptability and implementation of the screener in pediatric settings.

## Introduction

A large body of research has demonstrated a relationship between Adverse Childhood Experiences (ACEs) and a range of mental, physical, and behavioral health outcomes (Felitti et al., 1998; Hughes et al., 2017). ACEs include family-level experiences of abuse, neglect, or household challenges (i.e., caregiver mental health or substance use issues, domestic violence, and caregiver incarceration; Centers for Disease Co, 2016) that an individual experiences before the age of 18. Other social drivers of health (i.e., housing instability, food access) or community-level experiences of adversity (i.e., bullying, discrimination, foster care-involvement, community violence; ACEs Aware n.d.) expand beyond the initial ACEs screening and research. Based on growing evidence, scholars have pointed out that systemic-related adversities, such as institutional and cultural racism, can also contribute to ACEs (Shonkoff et al., 2021).

Numerous studies have demonstrated that ACEs predict psychosocial and behavioral outcomes in adulthood, including a heightened risk of problematic drug and alcohol use, sexual risk-taking, violence-involvement, depressive symptoms, anxiety, panic, suicidal ideation, and other self-directed violence (Dube et al., 2002; Hughes et al., 2017; Petruccelli et al., 2019). ACEs have also been linked to serious long-term health outcomes, such as cancer, heart disease, respiratory disease, gastrointestinal diseases, and mortality (Hughes et al., 2017; Petruccelli et al., 2019). Research indicates a dose-response relationship, such that a higher number of ACEs increases the probability of negative mental and physical health outcomes (Petruccelli et al., 2019).

## ACEs Screening

Given the relationship between ACEs and various health conditions, there has been a growing movement to integrate routine ACEs screening in primary care settings (Rariden et al., 2021). Routine ACEs screening is still in the relatively early stages of implementation which has highlighted numerous areas for continued research (Rariden et al., 2021). Despite concerns about asking about traumatic experiences and patient comfort, studies have found that ACEs screening is acceptable in primary care settings (Gillespie & Folger, 2017; Rariden et al., 2021). Research has begun to uncover areas of focus to improve the feasibility and acceptability of these screenings, including concerns related to: lack of time, lack of knowledge on the topic of ACEs, hesitancy to discuss personal topics with families, and inadequate systems to address positive screening results (Kia-Keating et al., 2019; Rariden et al., 2021). Notable factors that relate to patient responses and increased acceptability include patients’ preferred location to complete the screening (e.g., in the waiting room, private exam rooms, before or after an office visit) and information provided to patients about the purpose of the screening (American Academy of Pediatrics, 2014; Center for Youth Wellness, 2018; Rariden et al., 2021).

Initial studies have found that the implementation of ACEs screening has resulted in minimal disruptions to office flow or visits, even for patients with high ACE scores (Gillespie & Folger, 2017; Rariden et al., 2021). Also, post-implementation, providers have expressed benefits of ACEs screening including improved provider-patient relationships and communication, more empathy toward patients, and enhanced integrated care services (Gillespie & Folger, 2017; Kia-Keating et al., 2019; Rariden et al., 2021). In addition, a systematic review across 13 studies found that offering trauma-informed training and education on ACEs screening increased provider awareness, understanding, and proficiency, and may be an important component for providers and clinics newly implementing screening workflows (Rariden et al., 2021).

### Pediatric ACEs and Related Life Event Screener (PEARLS)

Several screening tools exist to collect reports of ACEs during childhood. The original ACEs Study Questionnaire focused on three domains: abuse, neglect, and family challenges. Similarly, existing practices at the time of the PEARLS tool development used screeners focusing on specific ACEs categories such as child abuse (e.g. the Childhood Trauma Questionnaire (CTQ) or assessing trauma more generally (e.g. the Traumatic Events Screening Inventory) (Koita et al., 2018). Researchers at University of California, San Francisco (UCSF), UCSF Benioff Children’s Hospital Oakland, and the Center for Youth Wellness created the PEARLS tool to assess ACEs and related life events cumulatively and holistically (Koita et al., 2018). From the initial ACEs Study Questionnaire, the authors added a fourth domain of social drivers of health (i.e., the social context), which included food insecurity, housing instability, violence outside the home, and experiences of discrimination. These items were adapted from the Center for Youth Wellness ACEs Questionnaire (Center for Youth Wellness ACE-Questionnaire, 2015), World Health Organization ACEs International Questionnaire (World Health Organization, 2020), and Family Information and Navigation Desk (University of California San Francisco Benioff Children’s Hospital Oakland, n.d.). Furthermore, the authors amended existing items to reflect diverse familial situations and existing research that underscored their effects on child development; the original “incarceration” item was expanded to include experiences of foster care, immigration, and death of a caregiver (Turney & Wildeman, 2017; Zayas et al., 2015), and “divorce” was broadened to assess family cohesion as a whole (Fomby & Cherlin, 2007; Lee & McLanahan, 2015; Stannard et al., 2022).

The PEARLS tool was finalized into a 17-item ACEs screener, co-created with patient families and clinical practitioners, for use in primary care settings (Author citation). PEARLS includes two parts, where Part 1 screens for the ten original ACEs categories, and Part 2 evaluates the presence of additional adversities. Individuals sum their responses to each part, with a possible maximum score of 17. Internal consistency of PEARLS scores, measured via KR-20, which is a special case of Cronbach’s alpha, has been adequate to high (0.81 and 0.82; Author citation). Evaluation of the PEARLS tool has demonstrated validity in not only reporting accurate ACEs exposure but also associations with poorer child health. Previous studies have identified a relationship between higher PEARLS scores and poorer global executive functioning, poorer perceived child health overall, and greater odds of stomach aches (Thakur et al., 2020; Ye et al., 2023). Overall, PEARLS has demonstrated construct validity in identifying three domains of adversity: maltreatment, household challenges, and social context (Ye et al., 2023).

Given that the research is still in its nascent stage, there is a continued need to carefully examine the implementation, acceptability, and feasibility of ACEs screening in collaboration with key stakeholders, including providers and consumers. The California ACEs Learning and Quality Improvement Collaborative (Cal-QIC) is a collaborative effort to accelerate the development of best practices of ACE screening, implementation, and response in primary care delivered within Federally Qualified Health Centers (FQHCs) and Community Health Centers. Through Cal-QIC, the PEARLS tool was implemented broadly and provided an opportunity to gather additional feasibility and acceptability data across diverse clinical settings. As part of the effort to strengthen and operationalize ACEs screening and response, the current study used participatory co-design methods (described further in the section below), collaborating directly with community members to apply organizational, linguistic, and workflow recommendations and refine the PEARLS tool, with a final outcome of creating PEARLS v2.

## Methods

### PEARLS Version 2 Development

Results and details of the development of the original PEARLS tool have been presented elsewhere (Koita et al., 2018; Thakur et al., 2020; Ye et al., 2023). In implementing the PEARLS tool across primary care settings within which the authors work and collaborate, providers and patients provided clinical practice feedback that the wording, structure, and presentation of the PEARLS tool could be improved upon. As the PEARLS tool expanded into more primary care settings, the feedback was echoed, and the team at Cal-QIC identified the issue of updating the PEARLS tool as a priority. A working group was formed with aims to create a version that included a broader range of ACEs and was more comfortable and clear for patients. The group, led by a public health manager, represented several academic-medical institutions and included physicians, clinical psychologists, mental health specialists, experts in ACEs research, graduate students, clinical research coordinators, and patients. In 2021, this group met biweekly to review the content of the tool and develop an updated version according to previously received feedback.

The review process for the first version of the PEARLS consisted of three parts, including (1) gathering feedback from Cal-QIC, the Cal-QIC Patient Advisory Board, and professional translators at UCSF, (2) adapting the tool, and (3) assessing for acceptability. Based on this process, the team made the following changes to develop version two of the PEARLS tool (PEARLS v2): First, Part 1, Question #1 of the original tool (“Has your child ever lived with a parent/caregiver who went to jail/prison?”) was moved down to become Part 1, Question #9; given the sensitivity of this questionnaire, this change was done to be ease caregivers’ comfort as they began to answer. Second, questions containing multiple parts were organized into a singular question stem, followed by individual options (“a, b, c”). Reorganizing the screener in this way allowed the group to more clearly express which experiences each prompt asked about.

Third, an introduction was added to the front page that clarified the purpose of the PEARLS v2 tool in inquiring about early adversity in patients’ lives. Specifically, it highlights that the tool is being implemented as a new addition to clinic workflow to ask all patients about stressful experiences; it also affirms that providing this information will help providers better support caregivers and their children. It states that the survey is confidential and optional, with a specific note about the limitations of confidentiality related to mandated reporting requirements. The decision to include this introduction stemmed from patient feedback in which they shared their wish for providers to clarify the tool’s intention prior to completing it at the clinic.

Finally, a pediatrics strength-based domain was added to the PEARLS v2 to acknowledge the resilience and positive qualities of both the child and family answering the questionnaire. This was in response to feedback from aforementioned groups to include a strength-based assessment as part of the tool. Two questions were added under this section: “What are your child’s best qualities?” and “What are things that help you (or your family) get through hard times (or cope with stress)?”. Selection of questions were informed by the growing literature on positive childhood experiences and in consultation with the multi-sectorial working group. Three bullet points were offered as space to answer these questions. The original domains (maltreatment, household challenges, and social context) were retained. For the Spanish translation of PEARLS v2, Cal-QIC UCSF Health equity interpreters offered edits for specific wording to be changed. Both these professional interpreters and Spanish-speaking members of the Cal-QIC Patient Community Advisory Board offered feedback on the screeners layout as well as a clause that patients will be able to discuss their responses with their providers given feedback that Spanish-speakers generally prefer the opportunity to explain their answers rather than yes/no questions alone. Additionally, three pediatricians offered feedback for the Spanish version that aligned with the English version changes, such as reordering the questions, simplifying the language to a 6th grade reading level, adding an introductory statement describing the purpose and confidentiality of the tool, and adding strength-based questions.

## Procedures

The team conducted semi-structured interviews with 13 caregivers of child patients at Federally Qualified Health Centers. The interviews were conducted via Zoom by researchers in English (*n* = 5) and Spanish (*n* = 8), depending on patient preference. The participants were recruited through provider referrals for adult (18+) caregivers of a child between the ages of 0–12 years old who had recently completed a well-child clinic visit. Among the caregivers who were interviewed, four family advisory board members at clinic site two were interviewed in the form of a focus group, and nine caregivers from clinic site one and two were individually interviewed. Seventeen caregivers were contacted, with 13 caregivers (76%) agreeing to participate.

The semi-structured interviews and family advisory board focus group were broken down into assessing the three parts of the PEARLS v2 survey: the introduction, the PEARLS Questions (Part 1 Questions 1–10 & Part 2 Questions 1–7), and the strength-based questions. The participating caregivers were presented with the screener tool in their preferred language, Spanish (*n* = 8) or English (*n* = 5). They were given time to read through each of the three parts one at a time. Interviewers offered to read aloud if they preferred or had trouble reading the screener but all participants preferred to read silently to themselves. Interviewers asked caregivers to notify them when they had completed reading each section so they were given as much time as necessary to review the items. They were asked about their perceived understanding and clarity of items, and comfort with the three parts. Additionally, they were asked if the PEARLS Questions matched their expectation from the introduction and if any changes could be made to improve the clarity of all parts of the PEARLS v2. Furthermore, participants were asked where in the clinic they would feel comfortable completing the screener, their preferred provider response after completing the screener, and how honest/comfortable they would be in completing the screener.

## Participants

Thirteen caregivers from two clinics participated in semi-structured interviews (*n* = 9) or a focus group (*n* = 4) that focused on their experiences with ACEs screenings and the PEARLS v2 tool. All caregivers identified as female and biological mothers of the child who had been screened. Caregivers’ ages ranged from 27 to 40. Caregivers were predominantly Latinx (85%; 7.5% Black; 7.5% white), and 61.5% spoke Spanish as their primary language (38.5% English-speaking). Among caregivers who reported their own ACEs (*n* = 5), the range was from 0 to 7. Child ACEs scores for those who reported ACES (*n* = 8) were either 0 (87.5%) or 2 (12.5%).

### Rapid Qualitative Analysis

Themes from the interviews and focus group were extracted using Rapid Qualitative Analysis (rapid QA), an iterative team-based approach that relies on group discussions to derive themes and preliminary conclusions from participant interviews (Hamilton, 2013, 2020; Schexnayder et al., 2023). Rapid QA is used in the context of health services research and implementation research due to the need for rapid turn-arounds and an increasing push for action-oriented, pragmatic approaches (Hamilton, 2013). Domain names were created to correspond with the interview protocol. For example, the interview question that stated, “How comfortable do you feel talking to your child’s provider about these issues?” was placed under the “Provider Comfort” domain. The 16 domains included: Introduction, PEARLS Questions - Comfort, PEARLS Questions - Clarity, PEARLS Questions - Helpful/Positive Feedback, What’s Missing, Comparison to PEARLS v1, Strength-Based Question Response, Strength-Based Question Order, Parent-Report, Provider Comfort, Honesty, De-Identified or Identified, Provider Resources/Response, Location/Time Preference, Other, and Quotations.

All interviews were recorded with permission and transcribed using Zoom auto-transcription services. Transcriptions were verified for accuracy in comparison to the video recording by members of the research team prior to analysis, and all transcripts were maintained in their original languages with quotations translated from Spanish to English for this manuscript by three bilingual research team members. The research team reviewed the transcripts and created a summary template for participant responses within each of the domains (see Appendix A: Table 2). A document was also created defining each domain and its scope (see Appendix C: Table 4). In order to assess for consistency across researchers, the coding team summarized the same two transcripts and reconvened to check for similarities and differences in coding. Once consistency had been established across researchers, the 10 transcripts were divided among the team, with at least two researchers coding each one. The research team met three times throughout this process to discuss any discrepancies in summaries and reach consensus. The summaries were then transferred into a Microsoft Excel data matrix; each row represented an interview transcript (i.e., an individual or focus group), and columns represented a domain (see Appendix B: Table 3). Finally, summaries were created across matrix domains, and these served as domain-specific codes. The research team then reviewed these codes to identify key themes from the interview transcripts.

## Results

### Screener Introduction and Implementation

Participants were asked about their perceptions of the introduction sheet that was added to PEARLS v2 to provide a rationale and understanding of the subsequent questions being asked. Participants reported overall that the introduction was clear and understandable and led them to expect screener questions about their child’s well-being, their exposure to stress and adversity, and/or the child’s context (i.e., social support systems, housing, income). As one participant noted, “Pues yo con la introducción, con la introducción, yo me siento muy bien. Siento que nos están apoyando y están este preocupándose por nuestro bienestar.” [“Well, I think with the introduction, with the introduction, I feel very good. I feel like they are supporting us, and they are concerned about our well-being.”].

A few participants noted their concerns about what would be done with the information after completion, considering the mention of mandated reporting in the introduction and the lack of specificity on what support services they may receive after completing the screener. Two participants noted that they would prefer not to spend too much time reading the screener instructions, and one noted that she usually skips directly to the questions when given a questionnaire like PEARLS v2. Some participants suggested that having a provider or staff member introduce and explain the screener verbally to them, in addition to the written description, would be beneficial to understanding the screener’s purpose and increase their comfort [See Table 1 for suggestions]. However, when compared to the original PEARLS, multiple participants noted that they found the PEARLS v2 introduction useful in adding a more in-depth explanation of the screener.

Caregivers were asked about their preferred location for completing the screener at the clinic. A majority of participants highlighted privacy and indicated that the exam room (e.g., as opposed to the waiting room) was the best option. One caregiver explained, “When you’re in the exam room, you have more of a private place to do it, and you would answer more confidently. Like sometimes you’re reading questions that make you nervous, and it would be better if you’re in the room alone.” Another participant also mentioned wanting to complete it while waiting for the provider to arrive in the exam room. However, others liked the efficiency of completing the screener in the waiting room, “Cuando yo llegue y me dan el documento, yo lo puedo llenar en la sala de espera y respondo rapidito. Y ya, cuando me llaman con el doctor ya yo lo entrego [When I arrive, and they give me the document, I can fill it out in the waiting room and answer quickly. And when they call me with the doctor, I hand it in].” Despite mixed responses on the exact location, participants generally appeared to prefer to complete the screener during a waiting period prior to seeing their provider.

### Screener Comfort and Clarity

Participants were asked about their comfort level and perceived clarity of the PEARLS v2 questions. They also provided direct feedback on how to improve the screening questions. Overall, the participants agreed that the questions were clear and matched their expectations from the introduction. As one of the participants noted, “I think all the questions are well explained.” They provided structural feedback to improve clarity, specifically to reduce the spacing between questions and answer boxes on the de-identified version. Additionally, they mentioned that in the identified version, all questions should have “YES” or “NO” options, with each part being summed up.

Some participants offered specific feedback on question phrasing to increase clarity and comfort, which can be seen in the Table 1 display of the original PEARLS item, PEARLS v2 item, and any participant suggestions for items. For example, in questions where there were multiple examples provided in subsections of the question (Part 1 Question #6 and #7; Part 2 Question #4), one participant recommended writing out the question fully and then listing examples to reduce confusion.

In terms of language, one caregiver suggested adding the word “intentionally” to Part 1, Questions #6 and #7. Additionally, another caregiver suggested that Part 1, Question #3 should include “another parent” as an option, seeming to suggest this participant had a concern that her answers would be interpreted as if the responding caregiver was personally involved in any items endorsed. This same caregiver also suggested that Part 1, Question #8 about sexual abuse be changed to, “Has your child ever experienced someone touching them without their permission?” Overall, the research team interpreted that these suggestions aimed at increasing comfort also reflected concerns about stigma or blame that arose for a few participants in reviewing the screener. However, these suggestions may create further confusion or misunderstanding of the question by other parents. For example, regardless of the intentionality of the action or who committed the action, PEARLS aims to screen for the child’s experience itself and does not aim to address the impact of that experience (i.e., post-traumatic stress). Additionally, in regards to the suggestion on screening for sexual abuse, it is important to note that minors (depending on age, capability, and state) are not legally able to provide sexual consent and the screener wishes to be clear in the language used to describe sexual abuse.

Participants also gave feedback on the language of the questions, mentioning that some of the questions were too descriptive in a violent and gruesome way [See suggested changes in Table 1]. They suggested that using more general language would increase comfort. For example, a participant suggested changing Part 1, Question #8 to avoid using the term “sexual abuse.” Moreover, they suggested that cultural differences in the Spanish language could change the connotation of words. In Part 1, Question #6, for example, “abofetear” (“to slap”) could be seen as more gruesome than “golpe” (“blow”). Another suggestion was using “etnicidad” over “etnia” for Part 2, Question #2; while both words translate to “ethnicity,” different vocabulary may be more commonly used in some Latinx communities. Lastly, a participant noted that in Part 2, Question #1, adding “bullying” to the Spanish-version as is present in the English-version would be more appropriate than “intimidación” considering “bullying” is commonly understood. Participants also felt that having a text box to expand on some of the questions or resources they would like to receive would be a good way to improve their comfort when answering the screener.

Several caregivers noted specific questions that may spark discomfort, such as Part 1, Question #3 [See Table 1]. One mother noted that the use of the word “humiliated” was too strong and may make people uncomfortable or fearful about suffering repercussions if they answered “yes.” She noted, “Pienso que son preguntas fuertes y muchas personas no contestan porque piensan que al contestar alguna pregunta de estas vayan a tener problemas o algo. Pero para mí está muy bien, muy claro y todo. [I think they are strong questions, and many people don’t answer because they think by answering one of these questions, they will have problems or something. But for me, it is very good, very clear and everything].” Another caregiver noted that Part 1, Question #6 [See Table 1] also had wording that could be perceived as jarring or unpleasant. They explained, “Well, in the country where I’m from, we don’t hear that word [beaten up]. That’s why, for me, that word is very harsh.” Participants also mentioned potential discomfort with Part 1, Question #8 [See Table 1] and Part 2, Question #3 (“Has your child ever had problems with housing?”). One participant mentioned cultural differences playing a role in the answers, “Es muy importante, especialmente porque la cultura pues de nosotros, de latino mexicanos, eh, cuando pides ayuda, automáticamente te nombran que eres frágil o no? [It’s very important, especially because, well, our culture, of Latino Mexicans, uh, when you ask for help, automatically they label you as fragile or not?]”.

In general, the participants reported they felt comfortable with the questions and the prospect of completing the screener at a doctor’s visit. One of our participants effectively summarized this sentiment as they shared, “I’m still dealing with my issues from my past, so you know you get kind of like, ‘Ooh, this uncomfortable question again,’ but you know I basically that’s just what I get. I get a little uncomfortable with it. But I expect these kinds of questions at a doctor’s office, and you know places like that, so.” Many caregivers reported an understanding of the importance of their providers knowing about life events that can impact their child’s physical and mental health and stated this would motivate caregivers to answer the screener.

Similarly to feedback regarding the introduction of the screener, most of the discomfort expressed by the caregivers was due to a lack of clarity on how the screener would be used to inform medical care and if there could be negative consequences from answering these questions. The participants mentioned that they would like to know upfront what knowledge, resources, and support they will gain, as well as what treatment plan will come from filling out the screener that could lead to direct benefits for the caregiver and child. They also wanted to be reassured that Child Protective Services (CPS) would not be involved. It was also noted that the screener may invoke feelings of blame in caregivers for their child’s ACEs, and therefore, efforts should be made to reduce shame and stigma about ACEs prior to and following the screener.

**Table 1:** Items displayed from original PEARLS, PEARLS version 2, and participant-suggested changes to items underlined

Item # (as it appears in Pearls Version 2)	Original PEARLS Item	Adapted PEARLS Version 2 Item	Potential Changes for Consideration
	*Part 1 Question #7* ¿Alguna vez su hijo(a) vio o escuchó que a su padre/madre/proveedor de cuidados le gritaran, maldicieran, insultaran o fuera humillado por otro adulto? O ¿Alguna vez su hijo(a) vio o escuchó que a su padre/madre/proveedor de cuidados lo abofeateran, patearan o le dieran una golpiza o lo hirieran con un arma?	*Part 1 Question #6* ¿Su hijo/a vio o escuchó a su padre/madre/cuidador *(marque sí*,* si es cierto para usted o su familia)* a. a. gritar, insultar o humillar a alguien que conoce? b. b. abofetear, patear, dar puñetazos, golpear o herir con un arma?	*Part 1 Question #6* ¿Su hijo/a vio o escuchó a su padre/madre/cuidador *(marque sí*,* si es cierto para usted o su familia)* a. a. gritar, insultar o humillar a alguien que conoce? b. b. golpear, patear, dar puñetazos, golpear o herir con un arma?
Part 2 Question #1	¿Alguna vez su hijo(a) vio, escuchó o fue víctima de violencia en su vecindario, comunidad o escuela? *(Por ejemplo*,* acoso o “bullying” con una victima especifica*,* agresión u otros actos violentos*,* guerra o terrorismo)*	¿Su hijo/a alguna vez vio, escuchó o fue víctima de violencia en su vecindario, comunidad o escuela? *(Por ejemplo*,* intimidación*,* agresión u otras acciones violentas*,* guerra o terrorismo).*	¿Su hijo/a alguna vez vio, escuchó o fue víctima de violencia en su vecindario, comunidad o escuela? *(Por ejemplo*,* bullying*,* agresión u otras acciones violentas*,* guerra o terrorismo).*
Part 2 Question #2	¿Alguna vez han discriminado a su hijo(a)? *(por ejemplo*,* lo(la) han molestado o hecho sentir inferior o excluido(a) por su raza*,* etnia*,* identidad de género*,* orientación sexual*,* religión*,* diferencias en el aprendizaje o discapacidades)*	¿Su hijo/a sufrió discriminación? *(Por ejemplo*,* que se lo moleste o se le haga sentir inferior o excluido/a por su raza*,* etnia*,* identidad de género*,* orientación sexual*,* religión*,* diferencias de aprendizaje o discapacidades).*	¿Su hijo/a sufrió discriminación? *(Por ejemplo*,* que se lo moleste o se le haga sentir inferior o excluido/a por su raza*,* etnicidad*,* identidad de género*,* orientación sexual*,* religión*,* diferencias de aprendizaje o discapacidades).*
Part 2 Question #4	Have you ever worried that your child did not have enough food to eat or that the food for your child would run out before you could buy more?	Are you currently or have you ever: (*Mark yes*,* if any apply)*: a. worried that the food for your child would run out before you got money to buy more? b.had the food you bought for your child not last and did not have money to buy more?	Are you currently or have you ever experienced any of the following? a.Worried that the food for your child would run out before you got money to buy more. b.Had the food you bought for your child not last and did not have money to buy more. (*Mark yes*,* if any apply)*
Part 3 Introduction	-	“Nos gustaría comprender más sobre las fortalezas y los recursos de su hijo/a y su familia”	“Nos gustaría entender más sobre la fortaleza y los recursos que necesita su hijo y su familia”
Part 3 Suggested Additional Question	-	-	“Is there any additional information you wish to provide that we can help you with?”
**Additional Feedback**			
	PEARLS v2 Introduction		Clarifying phrases for providers to use when presenting to caregivers verbally
Screener introduction	Many families experience stressful events in their lives. Over time these experiences may affect your child’s health and wellbeing. There are many things you can do and may already be doing to help. Because these events are so common, we are offering resources to all caregivers in our clinic on how to support your child and yourself. We are asking everyone these questions. Some people find that answering the questions gives you time to think about how certain experiences may be impacting your child’s health and what you can do to help. Your answers help us to support you and your child to be as healthy as possible. The survey is confidential and optional*. Your experiences are your own and no matter what you choose to share, we are here to help. *It is important for you to know though that if you tell us that your child is currently unsafe or being physically, verbally, or sexually hurt or neglected we may need to share this with your authorities.		• “Every patient receives this screener.” • “Your doctor may discuss the results with you during your visit if you wish.” • “The information you provide will be used to…” • “We offer the following resources and referrals…”

### Strength-Based Questions

Participants were asked to share their preferences about the content and order of Part 3, which included questions about their child’s best qualities and their family’s coping strategies during stress. Previous work to pilot the strength-based questions highlighted themes in the responses from caregivers about their child’s best qualities (i.e., personal characteristics, cognitive ability) and their coping strategies (i.e., leisure time, quality time; Woodfork et al., 2022). However, researchers also highlighted the need for future work to explore how these strength-based questions made caregivers feel (Woodfork et al., 2022). Nearly all caregivers reported feeling “good” after reading the questions. They liked the opportunity to list their child’s positive attributes and to reflect on their family’s ability to manage hard times. Still, one caregiver shared that answering questions about handling stress brought them back to a “negative reality.”

Most caregivers did not explicitly comment on the clarity of the items. However, two respondents described this part as “clear” and “understandable,” while one caregiver found the phrasing of the introduction to the strength-based questions confusing [See Table 1]. They recommended changing the introductory statement from “Nos gustaría comprender más sobre las fortalezas y los recursos de su hijo/a y su familia [We’d like to understand more about the strengths and resources of your child and your family” to “Nos gustaría entender más sobre la fortaleza y los recursos que necesita su hijo y su familia [We’d like to understand more about the strengths and resources that your child and family need].” This individual felt that using more straightforward language would facilitate the transition from the screening questions and convey this section’s objective better. Additionally, one participant suggested an open-ended question at the end of Part 3 for any other information a caregiver may want to provide in order to receive additional support or resources [see Table 1]. Participants were also asked to share their preferences regarding the order of the survey. Five participants (38%) preferred answering the strength-based questions before the screener questions (Parts 1 & 2); some caregivers felt uncomfortable with the transition between Part 2 and Part 3, and others felt that it would be better to feel the positivity of this section before answering the screener. In contrast, nine participants (69%) preferred answering the strength-based questions as the last section of the screener so that they could be left feeling optimistic.

### Caregiver Honesty

Caregivers were asked how comfortable they would feel answering questions honestly throughout the PEARLS v2. Responses were mixed, with some participants stating that they would feel comfortable completing the whole questionnaire, while one caregiver shared that they would not feel comfortable at all. Overall, answers varied depending on the question in the screener. One participant noted, “I would be comfortable and open, but you know, there’s intense questions. Most people, especially people in poverty, are going through something, uh, one of those things in the questionnaire, and they probably feel uncomfortable because they don’t know what’s going to happen to their child.” Again, caregivers reiterated that they would feel comfortable answering questions if they were told beforehand what would happen with the answers they are sharing. The general consensus was that caregivers wanted reassurance that the information provided would help them better care for their children and not be used against them.

Despite noting areas where discomfort may arise, most caregivers reported that they would answer the screener honestly with no reservations, given the importance of the topic. One participant even mentioned that the questions were perfect to her, saying, “Bueno para mí, pues todo está perfecto. No tengo dudas de ninguno. [Well, for me, well, everything is perfect. I don’t have any doubts about anything].” Still, some participants did mention circumstances where they believe other parents would not answer honestly, most of which involved situations where caregivers feared potential consequences. For example, the participants noted that some caregivers may not honestly answer Part 2, Question #3 (“Has your child ever had problems with housing?”) due to fear of CPS being contacted. Others mentioned that caregivers who are actively hurting their children or have in the past may also answer dishonestly because they feel threatened. As one participant noted, “A person that is actually, you know, that might be hurting a child, or having trouble with their anger, I don’t think that they would answer these honestly, you know.” One caregiver also described hesitancy to answer Part 1, Question #1 (“Do you think your child ever felt unsupported, unloved, and/or unprotected?”) because she is unsure of her one-year-old’s experience of feeling supported.

In terms of preference regarding completing the screener in a de-identified format (i.e., providing a final sum total number of experiences) or an identified format (i.e., identifying each specific experience), participants reported mixed responses. While some caregivers expressed a preference for the de-identified format to increase comfort and honesty, others reported a preference for an identified format to better facilitate specific resources and referrals. Still, other caregivers reported either format was acceptable and it should be left to the caregiver’s personal preference.

### Caregiver Preferences Regarding Provider Response and Resources

Caregivers were asked about the desired and expected response they would receive from providers after completing the PEARLS v2 tool. Caregivers reported generally that it was important for their providers to know this information about children’s ACEs in order to better understand the context of the family and be able to offer tailored resources. As one caregiver noted, “Yo pienso que todo este tipo de encuestas este a mi me parece muy bien que nos tomen en cuenta para, pues si, para mejorar. Pienso los servicios que nosotros mismos recibimos, eh, a mi me parece muy bien. Por eso me gusta participar. Porque, pues, si puedo tender uno sea que mi voz escuche, pues pienso que puede ayudar y eso es lo que yo pienso a mi me gusta compartir mi experiencia. [I think that all of these types of surveys, to me, it’s great that they consider us to, well, yes, to improve. I think the services that we receive seem really good to me. That’s why I like to participate. Because, well, I can have my voice heard, so I think that can help, and that’s why I think I like to share my experience].”

Some caregivers reported a desire and expectation for their provider to have a follow-up conversation with them following the completion of the screener in order to contextualize what the findings meant for their child’s health and create a plan of action for resources and referrals. Even caregivers who reported a desire and expectation for a follow-up conversation, however, noted that the desire to discuss this information may vary by caregiver and family. One caregiver suggested there be an option to check a box on the screener if they desired an additional conversation with their provider. The majority of caregivers suggested that providers take a person-centered approach and determine what is appropriate on a case-by-case basis, being careful not to push the discussion past the caregiver’s comfort level. Regardless of the length of the conversation or when it takes place, the caregivers overall emphasized the desire and expectation that their provider create a safe place to be heard and take the time to understand the family’s needs and context. As one mother stated, “I would expect them to listen and actually understand what me or any parent would be trying to explain to them.”

In terms of time spent discussing the screener with their provider after completion, caregiver preferences ranged from 5 to 30 min. Most responses had the caveat that the time spent would depend on the caregiver’s willingness to speak and the severity of their answers. One participant noted, “Depende de cuánto tiempo la persona desee hablar del tema… Hay personas que se quieren desahogar con alguien y hablan y hablan y cuenta y cuentan. A veces me pasa a mí asi y pues dura la conversación, pero depende de la persona. [It depends on the amount of time the person wishes to talk about this topic… There are people who want to relieve themselves with someone, and they talk and talk and tell and tell. Sometimes it happens to me like this and well the conversation lasts, but it depends on the person].” Caregivers generally had a preference for discussing screener responses with their provider. As one caregiver noted, “I would like the doctor to offer the help if you need it.”

In addition to the need for a provider to build rapport, provide emotional support, and utilize clinical judgment in follow-up conversations, most caregivers suggested an expectation for practical and relevant referrals for a variety of services. Some caregivers mentioned specifically that, depending on which ACEs are endorsed, they would expect referrals to services to address issues like domestic violence, housing and income instability, trauma and child abuse, and individual or group mental health services. As one mother stated, “If I answer these questions and you don’t give me the resources, I will feel offended.” Overall, the provider-caregiver relationship and the provider’s practical and emotional response following the screening process appear critical to caregivers’ perceived satisfaction and support.

## Discussion

The current study used a participatory co-design approach to develop the PEARLS v2 to offer a more comprehensive, clear, and user-friendly ACEs screening tool for use in primary care settings. Semi-structured interviews with caregivers of child patients (ages 0–12 years old) were conducted in English and Spanish in two Federally Qualified Health Centers, to gather input and gather initial information related to acceptability. Thirteen caregivers participated, including nine individual interviews and four family advisory board members who provided their feedback in a focus group format. Themes from the interviews and focus groups revealed that most participants found PEARLS v2 to be acceptable, with individuals expressing comfort and clarity in completing the screening tool. Participants offered additional logistical, cultural, and privacy considerations based on the introduction of the screener, screener comfort and clarity, the strength-based questions, caregiver honesty, and provider responses and resources. The findings underscore the potential of PEARLS v2 as a practical and well-received screening tool for identifying ACEs, indicating its promise for more widespread use in primary clinic workflows.

Participant feedback revealed that having an introduction to the screening tool was generally helpful. Caregivers shared that the introduction was clear, digestible, and offered useful context about what they were going to be asked in the screener. Some participants noted that they may skip over the introduction or not fully understand it, so having a quick verbal introduction by a staff member or provider may be more beneficial than a written introduction. This aligns with previous research suggesting that having introductions to screeners is helpful, particularly developing scripts to discuss the screening with caregivers (Center for Community Health and Evaluation, 2019). As mentioned in Table 1, these suggestions expand or reiterate on ideas already mentioned within the written screener such as the universality of the screener, mandated reporting, and expected follow-up. In terms of location (e.g., waiting room, exam room) and time (e.g., before the provider arrives, during the appointment) to complete the screener, participants had mixed responses, but overall seemed to prefer to use the waiting period prior to seeing their provider and to complete it somewhere in privacy. Existing literature highlights the importance of offering referrals for individuals with high ACEs, and also acknowledges the challenge posed by disparities in access to behavioral health practitioners or social workers across different regions (Finkelhor, 2018; Kia-Keating et al., 2019).

In reviewing the screener, participants offered feedback regarding comfort and clarity, highlighting both strengths and areas for improvement. Caregivers generally agreed that the screener was an important tool to integrate into medical visits and appreciated its overall clarity. However, they suggested adjustments to address linguistic and cultural differences, as outlined previously in this article.

Primary discomforts raised by participants included concerns about future use of their answers and potential stigma. Some caregivers were uncertain about how their information would be used. Clarifying the purpose of the PEARLS v2 in primary care settings, using the updated tool’s introduction, can begin to address these concerns. Providers and medical teams should also outline the purpose of using this tool in their specific settings, both verbally while introducing the tool and in communication with patients afterwards. One participant suggested incorporating the word “intentionally” to certain questions, such as Part 1, Question #6 and #7, presumably in order to reduce feelings of stigma or blame for the parent. This suggestion arose from caretakers hoping to distinguish “things are done under anger or stress that [they] don’t intend.” Another participant suggested adding “another parent” to Part 1, Question #3, presumably also to reduce blame and stigma. It is important to recognize that the PEARLS tool aims to identify children’s experiences regardless of intentionality or the caregiver involved, acknowledging that the impact on the child may remain regardless of these factors. Additionally, these changes in language may create further confusion or misunderstanding of the question. Although suggestions like this were not widespread, this feedback does underscore the necessity to integrate strategies to mitigate feelings of blame and stigma while implementing PEARLS. Emphasizing the commonality of ACEs and the availability of resources to support caregivers, especially under stress, could enhance comfort and satisfaction with the screener. This information could be added to the introduction page, included in the instruction box of the screener, and explained verbally by staff. Furthermore, rephrasing questions to allow caregivers to mark off areas related to social determinants of health they are interested in receiving more support about, while not necessarily disclosing their full context for fear of response, could open up more avenues to secure services and resources. Adjusting the language in the questions, in addition to increasing clarity about the expected provider response in the introduction, would reportedly increase caregiver comfort when answering the questions.

Regarding the newly added Part 3 (strength-based questions), nearly all caregivers were satisfied with the language and focus of this section. With the exception of one individual, they appreciated the opportunity to discuss their children’s strengths and to reflect on their families’ resilience. While there were some linguistic suggestions for Part 3 [see Table 1], both suggestions shifted the focus from what the families already possess in terms of strengths to what they need and therefore may be more appropriate additions to Part 1 or 2 of the screener. Overall, these questions provided a supportive framework that caregivers felt acknowledged their positive attributes.

Caregiver honesty on screening tools has also been an area of interest in ongoing research about universal screenings considering potential concerns around caregiver stigma and discomfort (Schneider et al., 2021). The current study found that the additions to the PEARLS v2, including the introduction to the tool and the strength-based questions, may help reduce stigma and increase honesty. Additionally, caregivers suggested other methods to reduce stigma discussed above, such as a verbal introduction of the tool by staff to explain the purpose of it, the universal nature of screening, how the information will be used, and any potential follow-up they can expect from their provider. The majority of caregivers reported they would answer the screener honestly while a few expressed hesitancy based on lack of clarity with the question or expectations that other caregivers may have difficulty being honest with the screener. When asked about how completing the screener in a de-identified manner (i.e. reporting a total score rather than endorsing individual experiences) would affect honesty, some caregivers reported that honesty would increase with a de-identified version. However, others emphasized that, given the importance of the subject matter and the need for tailored referrals, an identified version would be preferred. As some caregivers suggested, it may be helpful to leave the option to complete the form in an identified or de-identified manner up to the individual caregiver.

Relatedly, caregivers strongly emphasized the importance of receiving an individualized and attuned response from their providers. While nearly all caregivers reported that they would expect a list of resources and referrals, some caregivers particularly emphasized the need for the referrals to be high-quality and tailored and that providers should follow-up to see if referrals were actually reached. This aligns with previous research emphasizing the importance of family-centered and trauma-informed practices from providers to support satisfaction and honesty with screening (Schneider et al., 2021). Caregivers in our study emphasized not only the need for tailored practical support in response to screening but also person-centered emotional support, fostered through a trusting and supportive relationship with their provider. In addition to the suggestion for a verbal staff introduction to the screener, taking time after the screener with the provider to reiterate the use and purpose of the screener, the prevalence of ACEs, and the available supports would reportedly contribute to caregiver satisfaction. The length of suggested time for this conversation varied by caregiver and some caregivers did not personally think the discussion was needed but thought the option should be available for all caregivers to have a follow-up discussion with their provider. Given limited provider time and the disparities in available resources families are seeking, continued research is needed to explore methods for workflow adaptation and capacity building to support families’ needs in response to continued screening. While the tool may be relatively ideal for a low resource setting due to its brevity and predictive ability, there is a challenge in having adequate resources and interventions to respond to the need uncovered. Community health workers, such as Wellness Navigators, can be crucial to addressing this need and facilitating this workflow as demonstrated by previous research (Barnett et al., 2020). Barnett et al. (2021) demonstrates through systemic modeling the potential heterogenous impacts of ACEs screening on behavioral health care delivery and highlights this as a needed area for continued research and interdisciplinary discussion. Ultimately when considering caregiver satisfaction and acceptability of ACEs screening, this study suggests that having available and tailored resources in response to screening is highly valued.

### Limitations

While this study adds to the literature by highlighting the acceptability and feasibility of a specific ACEs screening tool developed with participatory co-design, the research is not without limitations. This study only assessed caregiver acceptability of the tool in English and Spanish and therefore caregivers with other language preferences may offer other suggestions for improvement and acceptability. Continued research will be needed to address other cultural and linguistic barriers as the tool is translated to more languages to increase accessibility. The sample in this study all identified as female and biological mothers of the child being screened and therefore perspectives may vary if other caregivers (i.e., biological fathers, grandparents, adoptive or foster parents) were able to be included in this study. Only one child in the caregiver-child dyads involved in this study had an ACEs score higher than zero. It is feasible that responses may vary if the sample of caregivers had children with higher ACEs scores at the time they were interviewed.

### Future Directions

Future research should conduct similar interviews in the remaining fifteen languages the PEARLS tool is available in. The tool has been validated in English, Spanish, and Portuguese and while many other translations are available, they have not yet been validated. Given the limitations of translating written works from one language to another, it is essential that culturally reliable feedback is collected to help increase clarity and comfort of the PEARLS tool. An additional avenue of research is to dive in deeper to the linguistic and cultural difference within communities that speak the same languages. For example, during the interviews with Spanish speakers, regional differences of wording played a role in how certain words were received and understood. As many of our participants mentioned that a verbal introduction and explanation of the PEARLS tool would be preferable, a natural follow-up to this research would be to determine how medical staff members could best introduce the screening tool. Concurrently, more data is needed on the implementation efforts required to adopt the PEARLS v2 to a primary care setting, considering the suggestions included in this research. Elucidating the staffing, training, workflows, and communication needed to expand PEARLS to new settings will help expand the trauma-informed care efforts associated with adopting the PEARLS tool.

## Conclusion

This study highlights a sample of caregivers within Federally Qualified Health Centers who reported that the PEARLS v2 tool has a clear introduction page that matches the content of the questionnaire, as well as clear screening and strength-based questions that allow caregivers to reflect on stressors and positive attributes of their children’s lives. They would willingly fill out the PEARLS V2 screening tool and noted the importance of screening, but would be more comfortable if more clinic staff support was integrated into the screening process (i.e., verbal introduction on the tools use and purpose, follow-up conversations with providers, tailored recommendations). This study offers support for the face validity and acceptability of the PEARLS v2 tool, as well as suggestions for adaptations to be made both to the tool and clinic workflow to improve feasibility, clarity, and comfort.

## Figures and Tables

**Table 3:**
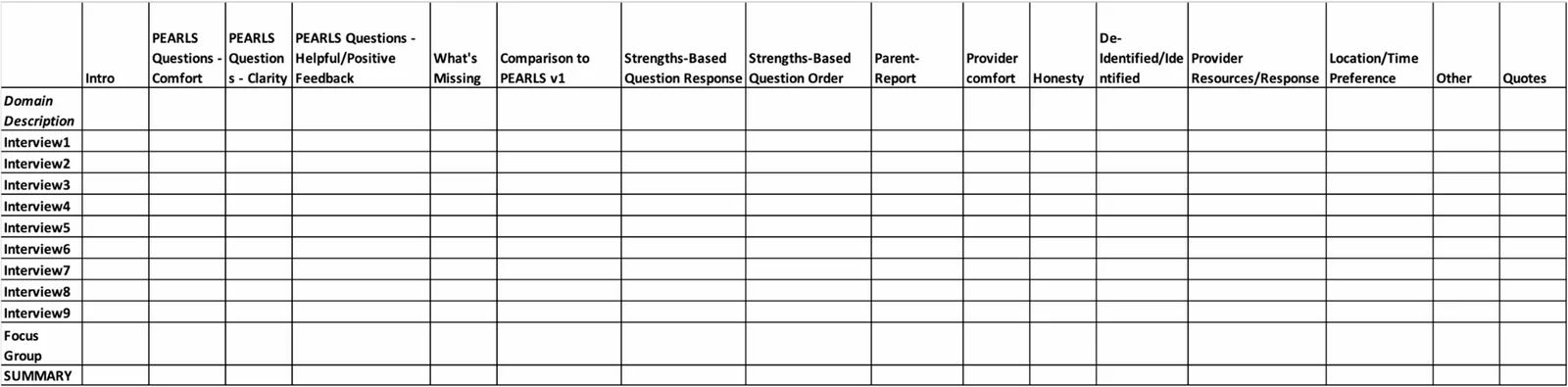
Data matrix template

## Data Availability

The data that support the findings of this study cannot be made publicly available to protect participant privacy. However, more information is available upon reasonable request.

## Appendix

**Table 2:** Transcript summary table template

Domain	Summary Example
Introduction	• Felt introduction was clear • Thought questions would be about stressful experiences
PEARLS Questions - Comfort	
PEARLS Questions - Clarity	
PEARLS Questions - Helpful/Positive Feedback	
What’s Missing	
Comparison to PEARLS v1	
Strength-Based Question Response	
Strength-Based Question Order	
Parent-Report	
Provider Comfort	
Honesty	
De-Identified or Identified	
Provider Resources/Response	
Location/Time Preference	
Other	
Quotations

**Table 4:** Domain name definitions

Introduction: Any thoughts, feelings, questions caregiver had in response to reading the PEARLS Introduction paragraph (Related to SSI Questions #1–2)
**PEARLS Questions - Comfort**: When a caregiver noted comfort or discomfort with a question. (Related to SSI Questions #3–4)
**PEARLS Questions - Clarity**: When a caregiver noted that a question was clear or unclear/difficult to answer in some way (Related to SSI Questions #3–4)
**PEARLS Questions - Helpful/Positive feedback**: When a caregiver noted that a question was helpful or expressed positive feedback about the inclusion of an item or screener overall. (Related to SSI Questions #3–4)
**What’s Missing**: When a caregiver noted there was an item or experience they thought should be included in the list OR when caregiver had questions about the tool that weren’t addressed in intro or questions themselves (Related to SSI Questions #3–4)
**Comparison to PEARLS v1**: When a caregiver was shown the original tool or offered feedback comparing new to old version (Related to SSI Questions #4)
**Strength-Based Questions Response**: When a caregiver reported feelings or thoughts they had following (Related to SSI Questions #5)
**Strength-Based Questions Order**: When a caregiver reported feedback about the when the Strength-Based questions should appear in the measure (before or after ACEs) (Related to SSI Questions #5)
**Parent-Report**: Feedback related to how a caregiver felt about completing the measure on behalf of their child (Related to SSI Questions #6)
**Provider comfort**: Feedback related to how a caregiver feels about discussing/completing the screen with pediatrician (Related to SSI Question #6 and 7a)
**Honesty**: Feedback related to how a caregiver feels about answering questions honestly (Related to SSI Questions #6)
**De-identified or Identified**: Feedback related to caregiver preferences for identifying specific experiences or a sum total of ACEs (Related to SSI Questions #7)
**Provider Resources/Response**: Feedback related to what a caregiver would expect/want from providers after screening (Related to SSI Questions #7b)
**Location/Time Preference**: Feedback related to caregiver preferences about how and where to complete screener, including how screener is introduced (Related to SSI Question #7c and #7d)
**Other**: Any other feedback not captured in above categories
**Quotations**: Here can mark down time-stamps and/or copy and paste impactful direct quotations
